# Monotone and fast computation of Euler’s constant

**DOI:** 10.1186/s13660-017-1507-8

**Published:** 2017-09-15

**Authors:** José A Adell, Alberto Lekuona

**Affiliations:** Departamento de Métodos Estadísticos, Facultad de Ciencias, Universidad de Zaragoza, Pedro Cerbuna 12, Zaragoza, 50009 Spain

**Keywords:** 11M35, 33F05, 60E05, Euler-Mascheroni constant, fast computation, infinite product, alternating zeta function, gamma process

## Abstract

We construct sequences of finite sums $(\tilde{l}_{n})_{n\geq 0}$ and $(\tilde{u}_{n})_{n\geq 0}$ converging increasingly and decreasingly, respectively, to the Euler-Mascheroni constant *γ* at the geometric rate 1/2. Such sequences are easy to compute and satisfy complete monotonicity-type properties. As a consequence, we obtain an infinite product representation for $2^{\gamma }$ converging in a monotone and fast way at the same time. We use a probabilistic approach based on a differentiation formula for the gamma process.

## Introduction

The Euler-Mascheroni constant *γ* was first introduced by Leonhard Euler (1707-1783) as

1 $$ \gamma = \lim_{n\to \infty } (H_{n}-\log n)=0.5772156\dots ,\quad H_{n}=\sum_{k=1}^{n} \frac{1}{k}, n=1,2,\ldots . $$

This constant appears in different mathematical subjects, such as number theory, special functions, random matrix theory, random permutations, and mathematical physics, among many others. We refer the interested reader to the survey paper by Lagarias [1].

As far as we know, two main types of computations of the Euler constant have been developed. The first one emphasizes the monotonicity of the corresponding convergent sequences, but the rates of convergence are relatively slow (polynomial rates). The second one emphasizes the speed of convergence (geometric rates), but looses the monotonicity in the approximation.

In this paper, we gather both points of view by providing approximating sequences that converge at the geometric rate 1/2 and satisfy complete monotonicity-type properties (monotonicity, convexity, etc.). In addition, such approximating sequences are easy to compute.

With respect to the first type of computations, we mention that Xu and You [2] and Lu et al. [3, 4] have used continued fractions to obtain monotone convergence to *γ*. For instance, it is shown in [2], Theorem 2, that

2 $$ \frac{C}{(n+1)^{11}}< \gamma -r_{n}< \frac{C}{n^{11}}, \qquad \frac{C^{\star }}{(n+1)^{12}}< r_{n}^{\star }-\gamma < \frac{C^{\star }}{n^{12}}, $$

where *C* and $C^{\star }$ are explicit constants, and $(r_{n})_{n\geq 1}$ and $(r_{n}^{\star })_{n\geq 1}$ are sequences involving the logarithm of a continued fraction, the first one being strictly increasing, and the second one strictly decreasing. Yang [5] has found the constants $(a_{i})_{0\leq i\leq s}$ for a given $s=0,1,\ldots $ such that

3 $$ H_{n}-\log \Biggl( n+ \sum_{i=0}^{s} \frac{a_{i}}{n^{i}} \Biggr) =\gamma + O \biggl( \frac{1}{n^{s+2}} \biggr) \quad \mbox{as }n\to \infty $$

is the fastest sequence converging to *γ*, giving in this way a constructive answer to a problem posed by Chen and Mortici [6]. It turns out that, for small values of *s*, the sequence on the left-hand side in (3) strictly increases to *γ*.

With regard to the second type of computations, K. and T. Hessami Pilehrood [7] have provided a rational approximation $p_{n}/q_{n}$ converging to *γ* subexponentially. In fact, these authors have shown that

4 $$ \frac{p_{n}}{q_{n}}-\gamma =e^{-4\sqrt{n}} \bigl( 2\pi +O \bigl( n^{-1/2} \bigr) \bigr) \quad \mbox{as } n\to \infty , $$

where

$$ q_{n}= \sum_{k=0}^{n} \binom{n}{k}^{2} k! ,\qquad p_{n}=\sum _{k=0}^{n} \binom{n}{k}^{2} k! (2H_{n-k}-H_{k}),\quad n=1,2,\ldots . $$

Exponential convergence to *γ* is possible at the price of using logarithms. In this respect, Karatsuba [8] showed that

5 $$ \gamma =1-\sum_{k=1}^{12n+1} \frac{(-1)^{k-1}n^{k+1}}{(k-1)!(k+1)} \biggl( \log n-\frac{1}{k+1} \biggr) + O \bigl( 2^{-n} \bigr) , $$

whereas Coffey [9] gave the formula

6 $$ \gamma =\frac{\log 2}{2}-\frac{2}{3\log 2}\sum _{k=1}^{\infty }\frac{1}{3^{k}} \sum _{j=1}^{k} (-1)^{j} \binom{k}{j}2^{j} \frac{\log (j+1)}{j+1}, $$

where the series in (6) has actually the geometric rate $1/3$. On the other hand, Sondow [10] obtained the expression

7 $$ \gamma =\frac{A_{n}-L_{n}}{\binom{2n}{n}}+ O \bigl( 2^{-6n} n^{-1/2} \bigr) \quad \mbox{as } n\to \infty , $$

where, for any $n=1,2,\ldots$ ,

$$ A_{n}=\sum_{i=0}^{n} \binom{n}{i}^{2} H_{n+i},\qquad L_{n}=2\sum _{0\leq i< j\leq n} \sum_{k=1}^{j-i} \frac{(-1)^{i+j-1}}{j-i} \binom{n}{i}\binom{n}{j}\log (n+i+k). $$

As mentioned before, the aim of this paper is to compute Euler’s constant in a monotone and fast way at the same time. To achieve this, we combine a formula obtained by Zhang and Williams [11] to compute Stieltjes constants (see also Coffey [9]) and a probabilistic perspective based on a differentiation formula for expectations of functions of the gamma process (see formula (20) in Section 3). More precisely, let

8 $$ \eta (z)=\sum_{m=0}^{\infty } \frac{(-1)^{m}}{(m+1)^{z}},\quad \Re (z)>0, $$

be the alternating zeta function. It was shown in Zhang and Williams [11], Theorem 4 (see also Coffey [9]) that

9 $$ \gamma =\frac{\log 2}{2}+\frac{\eta ' (1)}{\log 2}. $$

Our computation of $\eta '(1)$ is mainly based on the probabilistic representation given in Lemma 3.1 (Section 3).

We point out that some authors have introduced probabilistic tools to deal with different topics of analytic number theory. For instance, Sun [12] described Stirling series in terms of products of uniformly distributed random variables, Srivastava and Vignat [13] have given representations of the Bernoulli, Euler, and Gegenbauer polynomials in terms of moments of appropriate random variables, and Ta [14] has recently introduced a nice probabilistic approach to study Appell polynomials by connecting them to moments of random variables. Finally, fast computations of the Stieltjes constants using differentiation formulas for linear operators represented by stochastic processes can be found in [15] and the references therein.

## Main results

Denote by $\mathbb{N}$ the set of nonnegative integers, and let $\mathbb{N}_{+}=\mathbb{N}\setminus \{0\}$. The *m*th forward differences of any sequence $(v_{n})_{n\geq 0}$ of real numbers are recursively defined by $\Delta ^{0} v_{n}=v_{n}$, $\Delta ^{1} v_{n}=v_{n+1}-v_{n}$, $n\in \mathbb{N}$, and

$$ \Delta ^{m} v_{n}=\Delta ^{1} \bigl( \Delta ^{m-1}v_{n} \bigr) =\sum_{j=0}^{m} \binom{m}{j}(-1)^{m-j}v_{n+j},\quad m\in \mathbb{N}_{+}, n\in \mathbb{N}. $$

Let $n\in \mathbb{N}$. We consider the coefficients

10 $$ a_{n}(j)=\sum_{k=j}^{n} \binom{k+1}{j+1}\frac{1}{2^{k+2}},\quad j=0,1,\ldots ,n, $$

and define the following lower and upper approximants of $\eta ' (1)$:

11 $$ l_{n}=\sum_{j=0}^{n} a_{n}(j) \frac{(-1)^{j}\log (j+2)}{j+2} $$

and

12 $$ u_{n}=l_{n}+\frac{n+1}{2^{n+2}}\sum _{j=0}^{n+1} \binom{n+1}{j} \frac{(-1)^{j} \log (j+1)}{(j+1)j}, $$

respectively, where $\log 1/0:=1$. With these notations, we enunciate our first main result.

### Theorem 2.1

*Let* $n\in \mathbb{N}$. *Then*,

13 $$ \tilde{l}_{n}:= \frac{\log 2}{2}+\frac{l_{n}}{\log 2}< \gamma < \frac{\log 2}{2}+\frac{u_{n}}{\log 2}=:\tilde{u}_{n}. $$

*The sequences* $(l_{n})_{n\geq 0}$ *and* $(u_{n})_{n\geq 0}$ *satisfy the following complete monotonicity*-*type properties*

14 $$ (-1)^{m-1}\Delta ^{m} l_{n} \geq 0, \qquad (-1)^{m-1}\Delta ^{m} u_{n} \leq 0,\quad 1 \leq m\leq n+3. $$

*In addition*, *we have*

15 $$ u_{n}-l_{n}\leq \frac{n+1}{2^{n+2}}. $$

The sequences $(\tilde{l}_{n})_{n\geq 0}$ and $(\tilde{u}_{n})_{n\geq 0}$ in Theorem 2.1 provide monotone and fast computations of the Euler-Mascheroni constant *γ*. Moreover, such sequences are easy to compute. In this regard, let $S_{j}$, $j\in \mathbb{N}_{+}$, be a random variable having the negative binomial distribution with parameters *j* and $1/2$, that is,

$$ P(S_{j}=l)=\binom{j-1+l}{j-1} \frac{1}{2^{j+l}},\quad l\in \mathbb{N}. $$

The coefficients $a_{n}(j)$ in (10) can be represented as

$$ a_{n}(j)=\sum_{l=0}^{n-j} \binom{j+1+l}{j+1}\frac{1}{2^{j+2+l}}=P ( S_{j+2}\leq n-j ) ,\quad n\in\mathbb{N}, j=0,\ldots ,n. $$

Thus, the tail probabilities $a_{n}(j)$ can be precomputed, as done in many statistical packages, such as R. Finally, recall that a sequence $(v_{n})_{n\geq 0}$ is said to be completely monotonic if $(-1)^{m}\Delta ^{m} v_{n} \geq 0$, $m,n\in \mathbb{N}$. This is the reason why the inequalities in (14) are called complete monotonicity-type properties.

The approximating sequences to *γ* given in (2) and (3) are simpler to compute than those in Theorem 2.1. However, the sequences $(\tilde{l}_{n})_{n\geq 0}$ and $(\tilde{u}_{n})_{n\geq 0}$ in this theorem converge to *γ* in a much faster way and enjoy properties such as monotonicity, convexity, and so on. On the other hand, formulas (4), (5), (6), and (7) provide fast computations of *γ* at the price of loosing the monotonicity of the corresponding approximating sequences. Certainly, formula (7) computes *γ* in a faster way than that in (13). However, the sequences $(\tilde{l}_{n})_{n\geq 0}$ and $(\tilde{u}_{n})_{n\geq 0}$ in Theorem 2.1 are easier to compute than the main term in (7).

Denote

16 $$ P_{k}=\mbox{(even)}\prod_{j=0}^{k} (j+2)^{\binom{k+2}{j+2}},\qquad Q_{k}=\mbox{(odd)}\prod _{j=0}^{k} (j+2)^{\binom{k+2}{j+2}},\quad k\in \mathbb{N}, $$

where (even)∏ (resp. (odd)∏) means that the product is extended to those even (resp. odd) integers *j* running from 0 to *k*. As a consequence of Theorem 2.1, we give the following:

### Corollary 2.2

*We have the infinite product representation*

$$ 2^{\gamma -\log 2/2}=\lim_{n\to \infty } \prod _{k=0}^{n} \biggl( \frac{P_{k}}{Q_{k}} \biggr) ^{1/(k+2)2^{k+2}}, $$

*where* $P_{k}/Q_{k}>1$, $k\in \mathbb{N}$.

Guillera and Sondow [16], Example 5.8, have obtained the product formula

17 $$ e^{\gamma -\log 2/2}=\lim_{n\to \infty } \prod _{k=0}^{n} \biggl( \frac{Q_{k}^{\star }}{P_{k}^{\star }} \biggr) ^{1/(k+3)}, $$

where

18 $$ P_{k}^{\star }=\mbox{(even)}\prod _{j=0}^{k+2} (j+1)^{\binom{k+2}{j}},\qquad Q_{k}^{\star }=\mbox{(odd)}\prod_{j=0}^{k+2} (j+1)^{\binom{k+2}{j}},\quad k\in \mathbb{Z}_{+}. $$

However, the rate of convergence in Corollary 2.2 is faster than that in (17).

## Auxiliary results

Let $(X_{t})_{t\geq 0}$ be a gamma process (see Çınlar [17], pp.279-281), that is, a stochastic process starting at the origin, having independent stationary increments, and such that for each $t>0$, the random variable $X_{t}$ has the gamma density

19 $$ \rho _{t} (\theta )=\frac{1}{\Gamma (t)} \theta ^{t-1} e^{-\theta },\quad \theta >0. $$

On the other hand, let *V* and *T* be two independent random variables such that *V* is uniformly distributed on $[0,1]$ and *T* has the exponential density $\rho _{1} (\theta )$ defined in (19). We assume that *V* and *T* are independent of the gamma process $(X_{t})_{t\geq 0}$. Finally, let $f:\mathbb{R}_{+}\to \mathbb{R}$ be a differentiable function such that $f_{\star }(t):=Ef(X_{t})<\infty $, $t\geq 0$, where *E* stands for mathematical expectation. It has been shown in [18], Theorem 7.1, that

20 $$ f_{\star }' (t)=Ef ' (X_{t}+VT),\quad t\geq 0. $$

This formula can be applied to the problem at hand as follows.

### Lemma 3.1

*Let* *η* *be the alternating zeta function*. *Then*,

21 $$ \eta ' (1)= Eg(X_{1}+VT), $$

*where the function* *g* *is defined in* $\mathbb{R}_{+}$ *as*

22 $$ g(x)=\sum_{k=0}^{\infty } \frac{k+1}{2^{k+2}} \bigl( 1-e^{-x} \bigr) ^{k} e^{-x}=\frac{1}{4}-\sum_{k=0}^{\infty } \frac{k}{2^{k+3}}\bigl(1-e^{-x}\bigr)^{k+1}. $$

### Proof

By (19) the Laplace transform of $X_{t}$ is given by

23 $$ Ee^{-\lambda X_{t}}=\frac{1}{(\lambda +1)^{t}},\quad \lambda \geq 0, t\geq 0. $$

Thus, interchanging the sum with expectation, from (8) we have

24 $$ \eta (t)=\sum_{m=0}^{\infty }E \bigl( -e^{-X_{t}} \bigr) ^{m}=Ef(X_{t}),\quad t\geq 0, $$

where

25 $$ f(x)=\frac{1}{1+e^{-x}}=\sum_{k=0}^{\infty } \frac{(1-e^{-x})^{k}}{2^{k+1}},\quad x\geq 0. $$

Therefore from (20) and (24) we have

$$ \eta '(1)= Ef' (X_{1}+VT). $$

This shows (21), since $f'=g$, as follows from (25). Finally, the second equality in (22) follows by calculus. The proof is complete. □

In view of Lemma 3.1, we define, for any $n\in \mathbb{N}$ and $x\geq 0$,

26 $$ L_{n}(x)=\sum_{k=0}^{n} \frac{k+1}{2^{k+2}} \bigl( 1-e^{-x} \bigr) ^{k} e^{-x},\qquad U_{n}(x)=\frac{1}{4}-\sum _{k=0}^{n-1}\frac{k}{2^{k+3}} \bigl( 1-e^{-x} \bigr) ^{k+1}. $$

It can be checked from (26) that

27 $$ L_{n}(x)=U_{n}(x)-\frac{n+1}{2^{n+2}} \bigl( 1-e^{-x} \bigr) ^{n+1}. $$

Such partial sums allow us to give the following probabilistic representations of the sequences $(l_{n})_{n\geq 0}$ and $(u_{n})_{n\geq 0}$ respectively defined in (11) and (12).

### Lemma 3.2

*For any* $n\in \mathbb{N}$, *we have*

$$ l_{n}= EL_{n}(X_{1}+VT),\qquad u_{n}=EU_{n}(X_{1}+VT). $$

### Proof

By Fubini’s theorem the Laplace transform of *VT* is given by

28 $$ Ee^{-\lambda VT}=E \frac{1}{\lambda V +1}=\frac{\log (\lambda +1)}{\lambda },\quad\lambda \geq 0. $$

Since $X_{1}$ is independent of *VT*, from (23) and (28) we have

29 $$\begin{aligned} EL_{n}(X_{1}+VT)&= \sum_{k=0}^{n} \frac{k+1}{2^{k+2}}\sum _{j=0}^{k} \binom{k}{j}(-1)^{j} Ee^{-(j+1)(X_{1}+VT)} \\ &=\sum_{k=0}^{n} \frac{k+1}{2^{k+2}}\sum _{j=0}^{k} \binom{k}{j} \frac{(-1)^{j} \log (j+2)}{(j+2)(j+1)}=l_{n}, \end{aligned}$$

where the last equality follows from (10) and (11) after interchanging the order of summation. Similarly,

$$ E \bigl( 1-e^{-(X_{1}+VT)} \bigr) ^{n+1}=\sum _{j=0}^{n+1}\binom{n+1}{j}\frac{(-1)^{j}\log (j+1)}{(j+1)j}. $$

Therefore, the second equality in Lemma 3.2 follows from (12), (27), and (29). The proof is complete. □

Thanks to Lemma 3.2, the complete monotonicity-type properties of $(l_{n})_{n\geq 0}$ and $(u_{n})_{n\geq 0}$ are easily derived from the analogous properties satisfied by the sequences of functions $(L_{n}(x))_{n\geq 0}$ and $(U_{n}(x))_{n\geq 0}$.

### Lemma 3.3

*Let* $m\in \mathbb{N}_{+}$ *and* $n\in \mathbb{N}$ *with* $m\leq n+3$. *Then*,

$$ (-1)^{m-1}\Delta ^{m} L_{n}(x)\geq 0,\qquad (-1)^{m-1}\Delta ^{m} U_{n}(x)\leq 0,\quad x\geq 0. $$

### Proof

Fix $x\geq 0$. For any $s\geq 0$, denote

30 $$ v_{n}(s)=(n+s)\tau ^{n}(x),\quad \tau (x)= \frac{1-e^{-x}}{2}, n\in \mathbb{N}. $$

Using induction on *m*, we can check that

$$ (-1)^{m}\Delta ^{m} v_{n}(s)=\tau ^{n} (x) \bigl(1-\tau (x)\bigr)^{m-1} \bigl( n+s-(n+s+m)\tau (x) \bigr) , \quad n,m\in \mathbb{N}, $$

thus implying that

31 $$ (-1)^{m}\Delta ^{m} v_{n}(s)\geq 0,\quad m,n\in \mathbb{N}, m\leq n+s, $$

since $0\leq \tau (x)\leq 1/2$. On the other hand, from (26) and (30) we have

$$ \Delta ^{1} L_{n}(x)=\frac{e^{-x}}{4} (n+2) \tau^{n+1}(x)=\frac{e^{-x}}{4} v_{n+1}(1). $$

Therefore, the first inequality in Lemma 3.3 follows from (31). Finally, from (26) and (30) we have

$$ \Delta ^{1} U_{n}(x)=-\frac{n}{4}\tau ^{n+1}(x)=-\frac{\tau (x)}{4}v_{n}(0). $$

This, together with (31), shows the second inequality in Lemma 3.3 and completes the proof. □

## The proofs

### Proof of Theorem 2.1

Let $n\in \mathbb{N}$. By (26) and Lemmas 3.1 and 3.2 we get

$$ l_{n}=EL_{n}(X_{1}+VT)< Eg(X_{1}+VT)= \eta ' (1)< EU_{n}(X_{1}+VT)=u_{n}. $$

This, in conjunction with (9), shows (13). On the other hand, let $m\in \mathbb{N}_{+}$ with $1\leq m\leq n+3$. By Lemmas 3.2 and 3.3 we have

$$ (-1)^{m-1} \Delta ^{m} l_{n}=E(-1)^{m-1} \Delta ^{m} L_{n}(X_{1}+VT)\geq 0. $$

The second inequality in (14) is shown in a similar way. Finally, we see from (27) and Lemma 3.2 that

$$ u_{n}-l_{n} =\frac{n+1}{2^{n+2}} E \bigl( 1-e^{-(X_{1}+VT)} \bigr) ^{n+1}\leq \frac{n+1}{2^{n+2}}. $$

The proof is complete. □

### Proof of Corollary 2.2

Let $k\in \mathbb{N}$. Using (16), (23), and (28), we can check that

$$ \begin{aligned} \log \frac{P_{k}}{Q_{k}} &=\sum _{j=0}^{k} \binom{k+2}{j+2}(-1)^{j} \log (j+2) \\ &=(k+2) (k+1)E \bigl\{ \bigl( 1-e^{-(X_{1}+VT)} \bigr) ^{k} e^{-(X_{1}+VT)} \bigr\} >0, \end{aligned} $$

which implies that $P_{k}/Q_{k}>1$. Therefore, for any $n\in \mathbb{N}$, we have

$$ \log \prod_{k=0}^{n} \biggl( \frac{P_{k}}{Q_{k}} \biggr) ^{1/(k+2)2^{k+2}}=\sum_{k=0}^{n} \frac{1}{2^{k+2}}\sum_{j=0}^{k} \binom{k+1}{j+1}\frac{(-1)^{j}\log (j+2)}{j+2}=l_{n}, $$

where the last equality follows from (10) and (11). This, together with (13) and (15), shows the result. □

## References

[1] Lagarias JC (2013). Euler’s constant: Euler’s work and modern developments. Bull. Am. Math. Soc. (N.S.).

[2] Xu H, You X (2014). Continued fraction inequalities for the Euler-Mascheroni constant. J. Inequal. Appl..

[3] Lu D, Song L, Yu Y (2015). Some new continued fraction approximation of Euler’s constant. J. Number Theory.

[4] Lu D, Song L, Yu Y (2017). Some quicker continued fraction approximations and inequalities towards Euler’s constant. J. Number Theory.

[5] Yang S (2012). On an open problem of Chen and Mortici concerning the Euler-Mascheroni constant. J. Math. Anal. Appl..

[6] Chen C-P, Mortici C (2012). New sequence converging towards the Euler-Mascheroni constant. Comput. Math. Appl..

[7] Hessami Pilehrood Kh, Hessami Pilehrood T (2013). On a continued fraction expansion for Euler’s constant. J. Number Theory.

[8] Karatsuba EA (2000). On the computation of the Euler constant *γ*. Numer. Algorithms.

[9] Coffey MW (2010). The Stieltjes constants, their relation to the $\eta_{j}$ coefficients, and representation of the Hurwitz zeta function. Analysis.

[10] Sondow J (2003). Criteria for irrationality of Euler’s constant. Proc. Am. Math. Soc..

[11] Zhang NY, Williams KS (1994). Some results on the generalized Stieltjes constants. Analysis.

[12] Sun P (2005). Product of uniform distribution and Stirling numbers of the first kind. Acta Math. Sin. Engl. Ser..

[13] Srivastava HM, Vignat C (2012). Probabilistic proofs of some relationships between the Bernoulli and Euler polynomials. Eur. J. Pure Appl. Math..

[14] Ta BQ (2015). Probabilistic approach to Appell polynomials. Expo. Math..

[15] Adell JA (2012). Estimates of generalized Stieltjes constants with a quasi-geometric rate of decay. Proc. R. Soc., Math. Phys. Eng. Sci..

[16] Guillera J, Sondow J (2008). Double integrals and infinite products for some classical constants via analytic continuations of Lerch’s transcendent. Ramanujan J..

[17] Çınlar E (2011). Probability and Stochastics.

[18] Adell JA (2013). Differential calculus for linear operators represented by finite signed measures and applications. Acta Math. Hung..

